# Post-Vasectomy Semen Analysis: What’s All the Fuss about?

**DOI:** 10.3390/diagnostics14202275

**Published:** 2024-10-12

**Authors:** Kareim Khalafalla, Christopher Chee Kong Ho, Eric Chung, Widi Atmoko, Rupin Shah, Ashok Agarwal

**Affiliations:** 1 Department of Urology, Hamad Medical Corporation, Doha, Qatar; dr.kareim.moh2@gmail.com; 2 Department of Urology, College of Medicine, Qatar University, Doha, Qatar; 3 Global Andrology Forum, Moreland Hills, OH 44022, USA; 4 Department of Surgery, School of Medicine, Taylor’s University, Subang Jaya, Selangor, Malaysia; chrisckho2002@yahoo.com; 5 Department of Urology, Princess Alexandra Hospital, University of Queensland, Brisbane, Australia; ericchg@hotmail.com; 6 Department of Urology, Faculty of Medicine, Universitas Indonesia, Dr. Cipto Mangunkusumo Hospital, Jakarta, Indonesia; dr.widiatmoko@yahoo.com; 7 Department of Urology, Lilavati Hospital and Research Centre, Mumbai, India; rupinurvashishah@gmail.com; 8 Cleveland Clinic, Cleveland, OH, USA

**Keywords:** post-vasectomy semen analysis, vasectomy failure, contraceptive effectiveness, diagnostic guidelines, sperm clearance analysis

## Abstract

Vasectomy is a reliable male contraceptive method with a success rate exceeding 98%. Despite its efficacy, vasectomy is not foolproof, with potential early and late failures requiring careful postoperative monitoring via post-vasectomy semen analysis (PVSA). Published guidelines emphasize the necessity of conducting PVSA to ensure clinical sterility. Despite these clear guidelines, discrepancies in adherence and interpretation persist, with significant mismatches between guidelines and actual practice. Recent shifts in societal attitudes toward reproductive autonomy, spurred by significant political events and socioeconomic factors, have increased vasectomy rates, particularly among younger, childless men. This demographic change calls for enhanced PVSA compliance and clear communication about the non-immediate contraceptive effect of vasectomy. Home test kits have emerged as a convenient, though not always reliable, method for conducting PVSAs, which may require reevaluation in clinical practice. Given the variations across clinical guidelines and the challenges in achieving consistent PVSA outcomes, further research is needed to harmonize PVSA protocols across different health systems. PVSA is typically conducted between 8 and 16 weeks post-vasectomy, depending on the surgeon’s preference. Success is confirmed when a fresh, uncentrifuged sample exhibits either azoospermia, rare non-motile sperm (RNMS), or fewer than 100,000 non-motile sperm per milliliter. This effort will ensure that both patients and practitioners can rely on vasectomy as a safe and effective form of contraception. Effective patient counseling and strategic follow-up are crucial when it comes to managing expectations and ensuring compliance with post-vasectomy protocols, thereby minimizing the risk of unintended pregnancies post-procedure.

## 1. Introduction

### 1.1. Definition and Epidemiology of Vasectomy

Vasectomy is a secure and dependable surgical procedure designed for male contraception. It was initially introduced in 1899 for an inmate with a condition of excessive masturbation disorder; nearly a decade later, Belfield et al. advocated this method as a form of male contraception to achieve sterility in relation to family planning [1].

For many years, vasectomy has been a globally available option for couples who have completed their family and no longer wish to conceive children. It is recognized as one of the most effective methods of contraception, with success rates exceeding 98% [2,3]. Due to the ease with which vasectomy can be performed either in a medical office or day surgical unit, its popularity has grown steadily over the years. Presently, it is estimated that up to half a million vasectomies are performed annually in the United States alone [4]. However, only less than 3% of couples globally rely on vasectomy as a contraceptive method provided by their partners [5].

Cultural beliefs greatly impact the decision to undergo vasectomy, influencing both its acceptance and uptake across different regions. Religious views on contraception often create stigma, with some groups considering vasectomy unnatural or morally wrong, which deters individuals from pursuing the procedure. Additionally, misinformation, especially in rural areas, can foster misconceptions about vasectomy’s effects on masculinity and sexual performance, complicating informed decision-making. Social norms and peer influences also play a role, as men may feel pressured to adhere to traditional expectations of fatherhood, affecting their choices about permanent contraception. Overall, cultural attitudes shape perceptions of vasectomy, underscoring the importance of targeted education and community engagement to overcome barriers and encourage informed decisions [6,7].

### 1.2. Increased Rates of Vasectomy

In July 2022, the reproductive landscape in the United States underwent a significant transformation following the Supreme Court’s decision to overturn Roe v. Wade. This landmark ruling has provided federal protection for abortion rights since 1973 [8]. This judicial reversal not only impacted women’s health rights but also had a profound effect on men’s choices concerning their reproductive responsibilities and options. The decision and its impact on the current sociopolitical climate and economic considerations catalyzed a surge in interest among men, especially those who are young and have not yet started a family, to seek more permanent forms of contraception, such as vasectomy [9]. Press and Broadcasting networks across the country reported that increasing numbers of both single men and those with a partner have been opting for vasectomy as a solution. Additionally, a Google Trends analysis on contraceptive interests revealed a significant rise in searches for different contraception methods, with vasectomy experiencing the highest increase of 7.14 times within just 72 h following the verdict [10,11,12,13].

Healthcare providers have noted that the inquiries about and appointments for vasectomies have spiked, reflecting a growing demand for reliable, long-term solutions to avoid unintended pregnancies, particularly in a new legal environment that might restrict access to other forms of reproductive healthcare.

### 1.3. Techniques of Vasectomy and Vasectomy Failure

Over the years, numerous techniques for isolating and occluding the vas deferens during vasectomy have been developed and refined. Both American and European guidelines provide various vas occlusion methods, without endorsing one technique as superior to others. These methods include mucosal cautery, fascial interposition, ligatures or clips, vas excision, open-ended vasectomy, and non-divisional vasectomy with extended electrocautery. The selection of a particular surgical method, or a combination of methods, is primarily based on the surgeon’s experience, technical skill, and personal preference to ensure optimal success [14,15].

While vasectomy is highly effective, it does not guarantee a 100% success rate in clinical sterility, especially in the early postoperative period. Vasectomy failure can be classified as either early or late, depending on the time elapsed since the procedure and whether the postoperative semen analysis confirmed sperm clearance. Vas recanalization following a vasectomy is considered a form of vasectomy failure, with several contributing factors. Early vasectomy failure has been reported in 0.2–5% of cases [16,17]. Several factors contributing to early failure include the surgeon’s technique, incorrect vas identification, and duplicated vas deferens. Various studies have noted a decreased incidence of early surgical failure with the use of fascial interposition during vasectomy [18,19,20,21]. Additionally, some authors suggest that applying cautery to the vas inner lumen on both ends can further reduce the incidence of early failure [22], while others commented that the length of the excised vas segment is an essential factor in preventing recanalization [23].

A duplicated vas deferens is a rare entity that occurs in 0.05% of the population [24] and tends to occur in isolation without association with other congenital anomalies [25]. These rare vas anomalies may be discovered during a genital examination or incidentally during inguinal surgeries such as varicocelectomy, hernia repair, vasectomy, or orchiopexy [24,26]. Duplicated vas deferens can be a cause of early vasectomy failure, necessitating readmission and resterilization [27,28].

Regarding late failure rates, it has been reported that approximately 1 in 2000 men who have undergone vasectomy may experience spontaneous delayed recanalization [29], despite the utilization of various techniques for occluding the vas deferens and confirmation of sperm absence in post-vasectomy semen analysis (PVSA) [14].

Specific precautions need to be taken, and measures should be implemented to confirm that vasectomy has been successful. Preoperative counseling plays a crucial role in this process, ensuring that all necessary details are thoroughly discussed with the couple seeking the procedure. This includes refraining from ejaculation in the first week to allow tissue healing and the use of other contraceptive methods until the success of the procedure has been confirmed. Moreover, men are counseled to ejaculate frequently after the first week post-operation to clear the pipeline of any sperm due to the possibility of the presence of motile sperm in the vasa, even after the procedure has been performed. There is no consensus on the exact number of ejaculations required during the early period, according to various studies. However, most authors suggest 20 or more ejaculations [14,30,31].

The American Urological Association (AUA) vasectomy guidelines have defined vasectomy failure as the presence of any motile sperm in the 6-month PVSA. They also added that “If >100,000 non-motile sperm/mL persist beyond six months after vasectomy, then trends of serial PVSAs and clinical judgment should be used to decide whether the vasectomy is a failure and whether repeat vasectomy should be considered. Expert Opinion” [14].

Our current commentary aims to elucidate the importance of post-vasectomy semen analysis, its indications, the consensus on different interpretations of the results, and strategies for effectively managing challenging cases. We will explore the controversies surrounding the timing of post-vasectomy semen analysis, the threshold for considering a successful vasectomy, and the varying guidelines on the number of clear samples required. Additionally, we will discuss how the presence of rare sperm influences decision-making and provide insights into the latest techniques for identifying and addressing recanalization. The paper also highlights best practices for counseling patients about potential failures and the subsequent steps to ensure long-term efficacy.

## 2. Discussion

### 2.1. The Controversy over PVSA

The vasectomy guideline statements from the AUA underscore the necessity of PVSA to verify sterility. PVSA is typically conducted between 8 and 16 weeks post-vasectomy, depending on the surgeon’s preference. Success is confirmed when a fresh, uncentrifuged sample exhibits either azoospermia, rare non-motile sperm (RNMS), or fewer than 100,000 non-motile sperm per milliliter [14]. Other society guidelines may vary in their recommendations regarding the timing of PVSA, the number of samples required to confirm success, whether the sample should be centrifuged or not, and the definition of special clearance in the event of the presence of more than 100 non-motile sperm (Table 1) [15,32,33].

After the release of the latest AUA guidelines for special clearance of PVSA, several authors studied the decreased PVSA required after implementing the special clearance parameters. Bieniek JM et al. conducted an investigation of 111 patients who underwent a PVSA, and sperm was detected in 40 of these patients. Of these, 38 patients, representing 97.4%, were deemed to be clear based on the latest clearance criteria, resulting in a cost saving of USD 2356 by eliminating the need for additional analyses [40].

A study by Manka et al., which analyzed over 4000 vasectomy cases from 2013 to 2017, revealed a significant mismatch between the 2012 AUA guidelines and urologists’ actual practices, particularly concerning the PVSA results which indicated the presence of more than 100,000 non-motile sperm. According to this study, 72% of the subsequent PVSAs were deemed unnecessary according to the AUA guidelines. This underscores both practitioners’ and patients’ efforts to confirm the success of vasectomy procedures, highlights the potential financial strain involved, and illustrates how various medical societies have modified their guidelines to address these concerns [41].

### 2.2. PVSA Compliance Rates and Impacting Factors

Several studies have proposed reasons for non-compliance, such as changes in partners or relationships, a strong belief in the procedure’s efficacy or confidence in the clinician, and insufficient communication regarding the importance of follow-up. Nonetheless, there is limited literature regarding the actual reason(s) for PVSA non-compliance and non-adherence [42,43,44].

In a separate study carried out at a family practice center, the medical records of 551 patients over ten years were examined to assess compliance rates, which were then correlated with patient age. The findings revealed that 58% of patients returned for the 6-week PVSA, 25% returned for the 3-month follow-up, and 8% attended the 1-year appointment. Additionally, the study observed a higher compliance rate among older patients, indicating a fear of unintended parenthood at an advanced age, which could impact the couple economically and might necessitate lifestyle adjustments [45].

Maatman et al. conducted a retrospective review of 1892 patients who underwent vasectomy. Only 33% of patients completed two PVSAs, and approximately 3% returned for their annual semen analysis to detect delayed recanalization. The authors hypothesized that the poor compliance rate could be attributed to various factors, including but not limited to changes in the treating physician or health plan, changes in the fertility potential of the partner, misunderstanding of post-vasectomy instructions, inability to perform a semen analysis, or fear of the results [46].

Moreover, in Diederichs et al.’s study investigating causes of non-compliance with PVSA, 99 telephone interviews were conducted with patients who did not complete their PVSA. The primary reasons for non-compliance were found to be patients being preoccupied with work (28.3%), having confidence that the procedure was successful (22.2%), or feeling that it was inconvenient to perform the PVSA (15.2%). The authors proposed future studies for causes of complaints in a way that will direct future patient and couple counseling [47].

In an intriguing effort to explore methods for increasing compliance with post-vasectomy semen analysis (PVSA), a team from New York divided their patients into two groups: those who received a semen analysis cup either during their postoperative follow-up and those who received a cup on the day of the procedure itself. The first group comprised 173 patients, while the second group comprised 197 patients. The researchers observed a higher likelihood of PVSA completion when the cup was provided on the same day as the vasectomy procedure compared to when it was provided during follow-up, with rates of 62.4% versus 49.7%, respectively [48].

Furthermore, Jacobsen et al. discovered no disparity in PVSA compliance and its timing when comparing patients with walk-in appointments between 8 and 16 weeks and those with scheduled appointments [49].

### 2.3. Home Test Kits and Location Convenience for PVSA

A key factor influencing compliance with PVSA is the availability and dependability of home testing kits, and whether the PVSA can be collected at home and mailed or must be conducted in-office.

Punjani and colleagues reviewed 364 vasectomies performed by a single surgeon, finding that 70% of the patients completed their PVSA in-office, while 30% used a HomeKit for the procedure. Compliance rates for both methods were statistically insignificant (59.6% for at-home testing versus 58.8% for in-office tests, *p* = 0.89). The authors concluded that there was no significant association or predictive measure between compliance and demographic factors such as the couple’s age, the number of children they had, their social habits (e.g., alcohol consumption and smoking), or the use of home testing kits [50]. Although their series had a 0% overall failure rate and no significant differences in compliance between in-office and home testing kits, the authors noted that home kits offered a convenient and viable alternative to standard testing. They highlighted the cost-effectiveness and accessibility of home kits, especially for individuals with limited insurance coverage, financial constraints, or discomfort with in-office testing [50].

With regard to collection convenience, Atkinson et al. compared the rates of compliance with PVSA between two groups: the postal strategy group (in which samples were collected at home and posted), which included 32,708 patients, and the non-postal group (from whom samples were collected at the lab), consisting of 26,192 patients. The findings revealed a significantly higher compliance rate in the postal group: 20.4% higher than in the non-postal group. Additionally, although the early detection rate of vasectomy failure was lower in the postal group, this difference was neither clinically nor statistically significant. Moreover, the rate of late failure detection was similar between the two groups [51].

On the contrary, Welliver et al. drew a completely different conclusion while studying 337 vasectomies performed between 2014 and 2020 by a single surgeon. One hundred thirty-six patients had home-based (HB) PVSA, while two hundred and one had lab-based (LB) PVSA. They reported a higher significant compliance rate with LB PVSA and found that HB tests, surgeon factors, and an increased number of children all contribute to reduced compliance with PVSA [52].

The current at-home semen analysis kits accessible to consumers in the US market include a variety of brands like Sperm Check Vasectomy by DNA Diagnostics Center and Swim Count by Motility Count APS in Commerce Township. However, the minimum sperm concentrations that can be detected by these kits are still above the threshold of 100,000 per milliliter, which may not assure the effectiveness of a vasectomy [53,54].

Alternatively, other postal kits permit patients to collect samples at home and mail them to specific certified labs for a detailed analysis, potentially offering a more definitive report. Examples of these kits include Overnite Male by the University of Illinois at Chicago, Male From Home, and post-vasectomy semen analysis [55,56,57].

## 3. PVSA Challenges

### 3.1. How Do We Deal with the Persistent Presence of Sperm in Post-Vasectomy Semen Analysis?

If any motile sperm is detected after 12 weeks or >20 ejaculations, then vasectomy is considered to have been unsuccessful. However, special clearance can be given for <100,000/mL non-motile sperm. Laboratories should routinely analyze samples within four hours of collection when checking for the presence of sperm. If non-motile sperm are detected, additional samples should be examined within one hour of collection [33].

The presence of any motile sperm in a PVSA at six months is deemed a definitive failure, warranting consideration of a repeat vasectomy. Conversely, detection of more than 100,000 non-motile sperm/mL at the six-month mark is seen as a potential failure. In such cases, the surgeon should evaluate the trends in PVSA results and apply clinical judgment to determine the next steps. Repeat vasectomy may be necessary at the surgeon’s discretion [14].

The following are the technical aspects of PVSA that should be considered, as recommended by Agarwal et al. [34].

#### 3.1.1. Specimen Collection

Patients should abstain from sexual activity for 2 to 7 days prior to sample collection, which can be conducted either through masturbation into a sterile container or using a specialized semen collection condom designed for analysis. It is important to use only specific non-spermicidal lubricants for collection, avoiding saliva or any other lubricants like oils. The collected sample should be delivered to the laboratory at the scheduled appointment time within one hour of collection. During transport, the sample must be maintained at a temperature between 20 °C and 37 °C.

#### 3.1.2. Macroscopic Examination of the Sample

Upon full liquefaction, the sample’s pH, volume, color, and viscosity are assessed. If the sample displays high viscosity, it may undergo enzymatic treatment using trypsin by incubating it for an extra 10 min at 37 °C, or it can be mechanically decreased using methods like pipetting.

#### 3.1.3. Microscopic Examination of the Sample

During this phase, the analysis is centered on detecting the presence of sperm within the sample. Six µL of the specimen is placed onto a fixed cell-counting chamber. At least two wet mounts are thoroughly examined for sperm using high-power magnification (200×). If sperm are found, an estimate of the sperm count is performed. This count is manually determined to see if it exceeds, meets, or falls below the threshold of 100,000 non-motile sperm/mL. Additionally, sperm motility is evaluated; if motile sperm are present, their motility is estimated and recorded as a percentage [58].

However, confirmatory tests and their timing are subject to clinical limitations, which can vary depending on the laboratory equipment and the technologist conducting the analysis. To address these challenges, proficiency testing should be carried out by an accreditation agency. Laboratory results must fall within ±2 standard deviations of the mean for all participating laboratories. Additionally, medical technologists undergo an annual competency assessment to ensure accurate performance of the PVSA test, using a standardized competency checklist. Their knowledge of the PVSA test and AUA criteria is evaluated through a quiz, and passing these assessments is required in order to obtain approval to conduct PVSA evaluations. Quality control measures are implemented daily for sperm count and motility assessment, ensuring that manual evaluations of the sperm concentration and motility remain within 20% of the values obtained by a computer-assisted semen analyzer in a normal sample [34].

## 4. Conclusions and Our Expert’s Comments

Given the variations across different guidelines, there is a pressing need for further research aimed at unifying the current approaches to interpreting results from post-vasectomy semen analysis. To enhance adherence to PVSA, it is crucial for the surgeon to communicate clearly before the procedure that vasectomy should not be considered an instant means of contraceptive-free (unprotected) sexual intercourse. Appropriate consultations should be scheduled to review the patient’s status and reinforce the message about the continued necessity of using alternative contraceptive methods until clinical sterility is confirmed or “special clearance” is achieved.

Furthermore, patients should be informed that the risk of pregnancy persists until the vasectomy has been confirmed to be successful, and having the patient’s partner attend clinic visits can help to emphasize the importance of using contraceptives during the interim period.

Another point to emphasize is that in cases where there is still motile sperm or >100,000/mL non-motile sperm after six months, it may be necessary to repeat the procedure. It is recommended that a repeat vasectomy be conducted under general anesthesia with proper scrotal exploration and excision of at least 1 cm of vas on each side, followed by fascial interposition, to minimize the possibility of a second failure. Finally, the routine practice of semen analysis after vasectomy is crucial. Variations in follow-up practices, driven by economic factors and by losing patients to follow-up in different regions, expose significant gaps in current healthcare delivery. The authors also recommend establishing long-term health monitoring for patients who have undergone a vasectomy, addressing both the physical and psychological aspects of the procedure. This can be achieved through in-person visits or telemedicine services to assess success, record failure rates, and identify pregnancy risks. Research in this area would be highly beneficial, as it has the potential to enhance health services and improve patient outcomes.

## 5. Clinical Cases

### 5.1. Laboratory Scenario

A patient undergoes a PVSA at 12 weeks and is found to have a sperm concentration of 2.20 M/mL with 6% motility, resulting in a failed test. A second PVSA is conducted four weeks later, showing a sperm concentration of 0.70 M/mL with the presence of rare motile sperm (RMS). A third PVSA, performed another four weeks later, indicates a sperm concentration of 0.30 M/mL, with RMS still present.

Answer: According to AUA, EUA, and BAUS guidelines, the third PVSA is considered to be a failure. However, the significant decrease in sperm count and motility suggests a potential progression towards azoospermia. The physician is informed of the results to provide appropriate counseling to the patient.

### 5.2. Clinical Scenario

A patient who underwent a vasectomy three years ago reports that his wife is now six weeks pregnant and claims that he was cleared by his surgeon through PVSA. How should this situation be addressed?

Response: A fresh semen analysis should be conducted, and the initial post-PVSA reports should be reviewed. If the current report shows motile sperm, but the previous PVSA indicated azoospermia or rare non-motile sperm (RNMS), the physician should explain that the pregnancy could be due to spontaneous recanalization. Even if no sperm are found in the current report, late recanalization is still a possibility. It is essential that patients are counseled before vasectomy about the 1 in 2000 risk of pregnancy following a vasectomy, even if the PVSA results are clear. Lastly, the patient can be informed about the option of genetic testing of the offspring to confirm paternity.

Key points:

1. Guidelines vary in terms of their recommendations and standard settings on vasectomy.
2. Vasectomy failure can be classified as either early or late, depending on the time elapsed since the procedure and whether postoperative semen analysis confirmed sperm clearance.
3. Success is confirmed when a fresh, uncentrifuged sample exhibits either azoospermia, rare non-motile sperm, or fewer than 100,000 non-motile sperm per milliliter.
4. Proficiency testing and competency assessment are vital to ensure quality control.

## Figures and Tables

**Table 1:** Current society guidelines for PVSA screening outcomes: [34].

Society Name Guidelines/Year	Abstinence/Ejaculations	Specimen Collection and Examination	Pass/Fail Criteria for PVSA	Comments
AUA/2012 (amended 2015) [14,35]	Advised at 8–16 weeks post-procedure	Analyze a fresh, single, uncentrifuged semen sample within 2 h of ejaculation	**Success:** Azoospermia or RNMS or ≤100,000 non-motile sperm/mL **Failure:** Presence of motile sperm or >100,000 non-motile sperm/mL	Clinical judgment is necessary if >100,000 non-motile sperm/mL persists beyond 6 months post-vasectomy. The vasectomy is deemed unsuccessful if motile sperm are detected after 6 months.
ABA, BAS, BAUS/2016 [33,36]	Conduct test 12 weeks after surgery and after at least 20 ejaculations	Routine analysis within 4 h; if non-motile sperm are detected, reanalyze additional samples within 1 h	**Success:** Azoospermia or <100,000 non-motile sperm/mL **Special Clearance:** Not granted if motile sperm are detected	Special clearance is only provided after two samples meet success criteria.
EAU/2012 [15,37]	Screening at 3 months post-procedure after at least 20 ejaculations	Semen analysis follows WHO 2010 guidelines	**Success:** Azoospermia or <100,000 non-motile sperm/mL	Persistent motile sperm beyond 6 months suggests the need for a repeat vasectomy.
CUA/2016 [38]	Semen analysis recommended 3 months post-procedure after 2–7 days of abstinence	Perform semen analysis on both whole unprocessed and centrifuged samples; sperm concentration measured at 400× magnification	**Success**: A single sample with azoospermia or two samples with <100,000 immotile sperm/mL **Failure:** Detection of motile sperm or >100,000 immotile sperm/mL	Evaluating two samples after the procedure offers a more reliable prediction of success than evaluating just one.
ASERNIP-S/2014 [39]	Semen analysis at 3 months post-procedure following at least 20 ejaculations	No specific collection guidelines mentioned	**Success:** Azoospermia or two consecutive tests showing <100,000 immotile sperm/mL at least 7 months post-procedure **Failure:** Presence of motile sperm—requires confirmation with a repeat test after 1 month.	The presence of immotile sperm is inconclusive; repeated tests are necessary every month until azoospermia or special clearance is achieved.

PVSA: post-vasectomy semen analysis, AUA: American Urological Association, ABA: Association of Biomedical Andrologists, BAS: British Andrology Society, BAUS: British Association of Urological Surgeons, EAU: European Association of Urology, CUA: Canadian Urological Association, ASERNIP-S: Australian Safety and Efficacy Register of New Interventional Procedures—Surgical, RNMS: rare non-motile sperm, WHO: World Health Organization.
